# The Clinical Implications of Tumor Mutational Burden in Osteosarcoma

**DOI:** 10.3389/fonc.2020.595527

**Published:** 2021-04-07

**Authors:** Lu Xie, Yufei Yang, Wei Guo, Dongxue Che, Jie Xu, Xin Sun, Kuisheng Liu, Tingting Ren, Xingyu Liu, Yi Yang, Tao Ji, Xiaodong Tang

**Affiliations:** ^1^ Musculoskeletal Tumor Center, Peking University People’s Hospital, Beijing, China; ^2^ The Division of Bioinformatics, Genetron Health (Beijing) Co. Ltd., Beijing, China

**Keywords:** tumor mutational burden, progression-free survival, overall survival, osteosarcoma, whole-exome sequencing

## Abstract

**Background:**

Osteosarcoma (OTS) is aggressive bone malignancy without well-recognized prognosis biomarker. Tumor mutational burden (TMB) has been proved as effective biomarker in predicting clinical outcomes in several cancer types. However, its prognostic value in OTS remains unknown. In this study, we aim to evaluate the implication of TMB in OTS patients.

**Methods:**

To depict the landscape of somatic mutations in OTS, we performed Whole-Exome Sequencing (WES) on 31 OTS tissue samples and corresponding White Blood Cells (WBCs) as matched control. TMB was calculated as the total number of somatic alterations in coding regions normalized to the per sequenced genomic megabase (~30.4Mb in WES). The prognostic values of TMB were evaluated by Kaplan-Meier methods and Cox regression models.

**Results:**

The median age was 16.0 years at diagnosis, and 54.8% of patients were male. The most common genetic alterations were mainly involved in cell cycle and DNA damage response and repair, including H3F3A, TP53, MYC, and CDKN2A/B. The median progression-free survival (PFS) was 775.5 days in TMB-High (defined as third quartile of TMB value, <2.565) versus 351 days in TMB-Low (<2.565). All patients with TMB-High are PFS-Long (>400 days), while 36.4% of all patients with TMB-Low were PFS-Long (*P*=0.003). TMB were significantly greater in PFS-Long than in PFS-Short (<400 days) (*P*=0.002). Moreover, the median overall survival (OS) was 1,307 days in TMB-High versus 672.5 days in TMB-Low. Furthermore, TMB-High group had significantly improved PFS (*P*=0.04) and OS (*P*=0.03).

**Conclusions:**

TMB-High can be used as prognostic marker for OTS. Our findings demonstrate that TMB may be helpful in combination with traditionally clinicopathologic risk factors to optimize risk stratification and guide treatment decisions.

## Introduction

Osteosarcoma (OTS) is a type of bone malignancy, predominantly occurring in children and adolescents as well as adults aged 40 years and over. Although neoadjuvant and adjuvant chemotherapy or surgical resection improves the 5-year survival rates from 20% to 70% since 1970s, the 5-year survival rate for patients with metastatic or relapsed OTS is only about 10%~30% (1, 2). Over the last 2 decades, chemotherapy is still the first line option for patients initially diagnosed as OTS (3). However, some patients with OTS don’t have durable response, leading to poor prognosis. Informative prognostic biomarker is in need for better patient’s stratification in terms of medication strategy.

Numerous studies have demonstrated that tumor mutational burden (TMB) is an independent biomarker to predict clinical outcomes. TMB is defined as total number of non-synonymous somatic mutations in coding areas of per tumor genomic megabase. High TMB has been reported to correlate with prolonged progression-free survival (PFS) and overall survival (OS) in metastatic triple-negative breast cancer (4), melanoma (5), NSCLC (6) and urothelial carcinoma (7). High TMB was associated with better 5-year PFS than that of low TMB in colorectal cancer (8). In contrast, high TMB had been shown to be associated with worse PFS in rhabdomyosarcoma (RMS) (9) and Ewing sarcomas (10). In addition, genomic alterations and allelic Imbalances were significantly associated with poor chemotherapy outcomes of patients with OTS (11, 12), while another study examined the genomic variation using a panel of 15 genes and found that the mutations did not predict the response to therapy (13). These controversial results suggest that it is necessary to systematically evaluate the prognostic role of TMB or gene alterations.

In this study, we characterized the genomic landscape of OTS through whole-exome sequencing (WES) on OTS tissue and corresponding WBC control samples. We found that the most frequent genomic alterations were mainly enriched in cell cycle and DNA damage response and repair (DDR). Moreover, we used the TMB as prognostic factor to evaluate the impact on clinical outcomes. The results suggested high TMB as an independently prognostic marker associated with improved survival in OTS patients.

## Material and Methods

### Ethics Approval and the Consent to Participate

This study was conducted after approval by the independent ethics committee and done in accordance with the ethical principles derived from the Declaration of Helsinki, International Conference on Harmonization Good Clinical Practice Guidelines and locally applicable laws and regulations. Informed consent was obtained from each patient. This trial was registered with ClinicalTrials.gov (NCT03336554).

### Samples and Patients

Thirty-one patients diagnosed as high-grade OTS and verified by two senior pathologists respectively from January 1st, 2014 to December 10th, 2018 were included. These patients were given neoadjuvant therapy followed by delayed definitive surgery and adjuvant chemotherapy according to the Peking University People’s Hospital-Osteosarcoma (PKUPH-OS) regimen, which included high-dose methotrexate, cisplatin, doxorubicin and ifosfamide. For patients with OTS progression after PKUPH-OS regimen, they accepted further chemotherapy including ifosfamide and etoposide or anti-angiogenesis tyrosine kinase inhibitors based on their individualized conditions.

All tumor tissues and WBC samples used in this study were collected in compliance with informed patient consents. This study was performed under a protocol approved from the Institutional Review Board (IRB) and in accordance with the China Common Rule.

### DNA Extraction, Next Generation Sequencing

Genomic DNA was extracted from fresh-frozen samples using QIAamp DNA Mini kit (Qiagen, 51306, Valencia, CA, USA) according to the manufacturer’ s protocol, and fragmented for constructing a library using KAPA Hyper Prep kits (KAPA, KK8504) and captured using the Agilent SureSelect XT Human All Exon v5 kit (Agilent Technologies, Santa Clara, CA, USA). On Illumina NovaSeq6000 platform, whole exome sequencing was performed on 31 tumors and corresponding WBC with average depth of 326x and 143x, respectively (**Supplementary Table S1**).

### Bioinformatics Analysis

Both tumor DNA samples and their matched normal DNA samples were analyzed according to standardized computational workflow: Raw sequence data (FastQ format) were trimmed and filtered using Trimmomatic 0.33 using the following parameters: 1) ILLUMINACLIP : TruSeq3-PE-adapter.fa:2:30:10:8:true; 2) TRAILING:3; 3) SLIDINGWINDOW:4:15; 4) MINLEN:36 (14). Paired-end clean reads were mapped to the human reference genome (hg19) using the Burrows–Wheeler Aligner (BWA, version 0.7.10-r789) by defaults parameters (15). Duplicate removal, local realignment, and base quality recalibration were performed using PICARD (http://broadinstitute.github.io/picard/, version 1.103) and the Genome Analysis Toolkit (GATK, version 3.1-0-g72492bb) (16). Somatic single nucleotide variations (SNVs) were called using Mutect (version 3.1-0-g72492bb) (17), using the Catalogue of Somatic Mutations in Cancer (COSMIC) v54 and dbSNP138 as reference sets of known somatic and germline mutations, respectively. Small indels were called using strelka (version 1.0.14) by defaults parameters with BAM as input (18). Effects of variants were annotated using Variant Effect Predictor (VEP, version 83, parameters “–everything, –fork 8, –buffer_size 1000”) (19). Each somatic variant was manually confirmed using Integrative Genomics Viewer (IGV, version 2.3.34) (20).

To identify significantly mutated genes (SMG), four algorithms were used to define significantly mutated genes (SMGs): MutSigCV (version 1.41; Benjamini-Hochberg false discovery rate q value <0.1) (21), MuSiC (v0.4; at least two FDR<=0.2) (22), OncodriveCLUST (V0.4.1; q-value <0.05) (23) and Oncodrive-FM (version 1.0.3; q-value <0.05) (24). SMG was defined if any gene was identified by more than one software.

Mutational signature analysis was performed using nonnegative matrix factorization (NMF) (25). 30 known signatures on COSMIC were used as reference. Somatic copy number variants (CNVs) were called using Control-FREEC (version 10.5) with default parameters (26). Recurrent copy number alterations were identified using GISTIC 2.0, q value < 0.05 was considered significant peaks (27).

TMB was calculated in somatic mutations in coding regions per megabase (MB). The OTS patients were divided into high TMB (TMB-High, defined as ≥2.565) and low TMB (TMB-Low, <2.565) groups by the third quartile of TMB value.

### Clinical Data

The collected clinical data includes age, gender, tumor anatomic location, histology subtype of tumors, tumor staging, surgical margins and follow-up information including death, last contact, vital status. Follow-up information was available for 30 patients since one patient data was missing.

### Statistical Analysis and Clinical Outcome Evaluation

PFS and OS were defined as the time from treatment initiation to the time of progression/last follow-up and death/last follow-up, whichever came first respectively. The association of TMB with PFS or OS were analyzed using univariate analysis and Cox proportional hazards regression analysis (multivariate analysis). Spearman’s correlation was used to analyze the correlation between TMB and age. Statistical analyses were carried out using R (v.3.4.1). *P* value < 0.05 was considered statistically significant.

## Results

### Sequence Data and Clinicopathological Characteristics

We examined 31 OTS tumor and corresponding WBC control samples by WES on Illumina NovaSeq6000 platform. The fraction of Q20 data quality is not less 90% (tumor range: 90.79%–98.77%, WBC ranger: 89.27%–98.69%). WES of target protein-coding sequence yielded a median of 327.34X tumor (range: 176.58X–435.87X) and WBC 143.67X (range: 87.25X–206.27X) distinct depth of average coverage. The median fraction of exonic bases in target regions with at least 10 unique reads of tumor and normal were 98.54% and 95.87%, respectively (**Supplementary Table S1**).

The demographics and clinicopathological data of 31 patients are summarized in **Table 1**. The median age was 16.0 years (range: 5.0–59.0) at diagnosis, and 54.8% of patients were male. Regarding histological subtypes, common subtypes (including osteoblastic, fibroblast and chondroblast) was the most frequent subtype (28/31, 90.3%). Giant cell malignancy transformation into osteosarcoma accounted for 6.5% (2/31). Small cell subtype was the least common subtype (1/31, 3.2%). The primary tumor sites included distal femur (13/31, 41.9%), distal radius (1/31, 3.2%), proximal tibia and femula (8/31, 25.8%), proximal femur (2/31, 6.5%), proximal humerus (4/31, 12.9%), pelvic (2/31, 6.5%), and cervical vertebra (1/31, 3.2%).

**Table 1:** Baseline patient and tumor characteristics.

Osteosarcoma N=31	
Age at diagnosis	
Median (Range)	16.0 (5.0–59.0 years)
Gender	
Male	17 (54.8%)
Female	14 (45.2%)
Histology Subtypes at diagnosis	
Common subtypes (Osteoblastic, Fibroblast, and Chondroblast types)	28 (90.3%)
Small cell subtype	1 (3.2%)
Giant cell malignancy transformation into osteosarcoma	2 (6.5%)
Primary site	
Distal femur	13 (41.9%)
Distal radius	1 (3.2%)
Proximal tibia and femula	8 (25.8%)
Proximal femur	2 (6.5%)
Proximal humerus	4 (12.9%)
Pelvic	2 (6.5%)
Cervical vertebra	1(3.2%)
Stage at diagnosis	
II A	1 (3.2%)
II B	22 (71.0%)
III	8 (25.8%)
Surgical margins achieved	
Wide/Radical	16 (51.6%)
Marginal	15 (48.4%)

### The Landscape of Somatic Mutation in OTS

**Figure 1:**
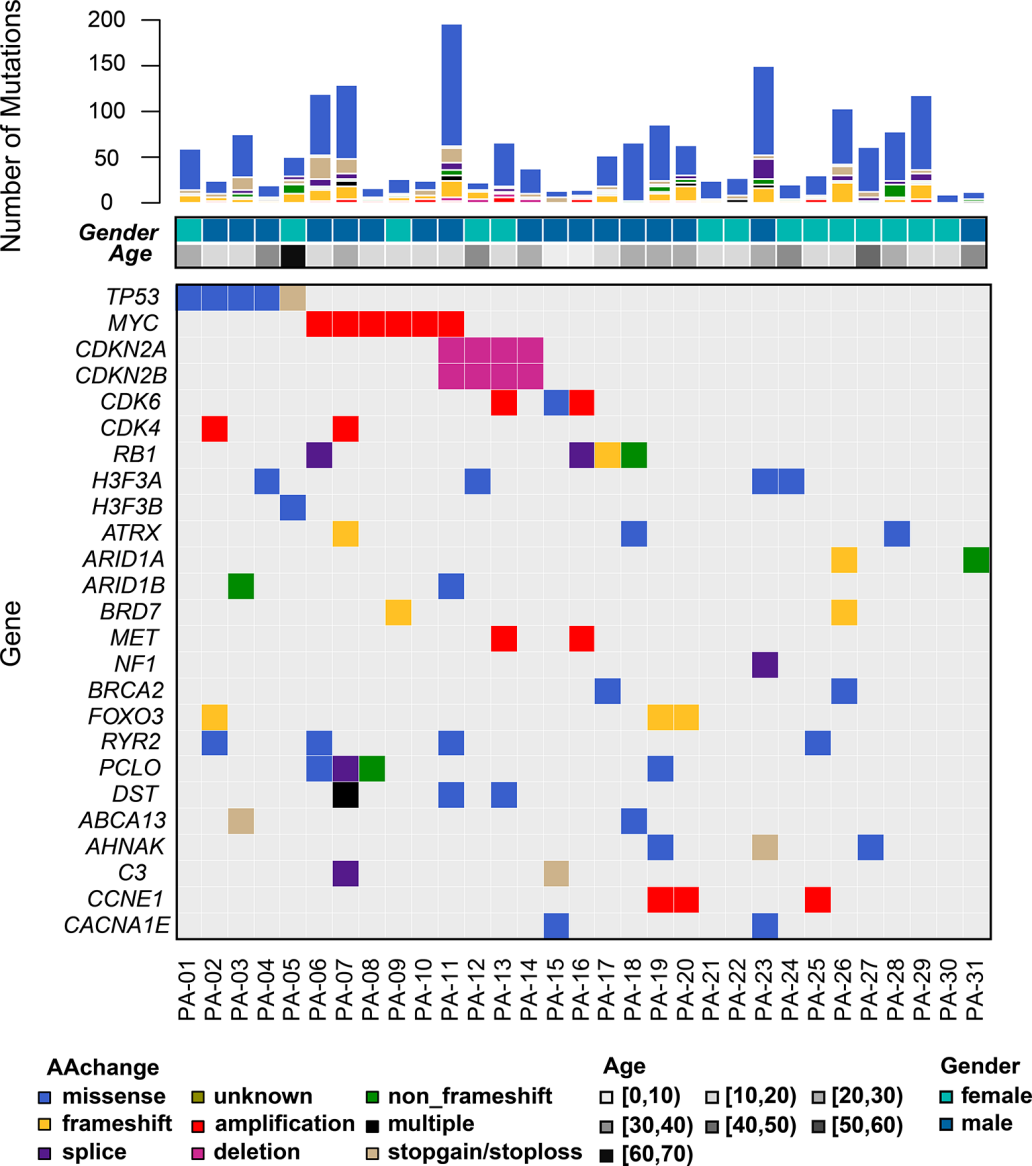
The mutational landscape of somatic alterations. Mutated genes are listed on the left-side. The genes with the mutations occurring ≥4 per sample and reported driver genes were included. Each column represents a sample. Top bar plot summarizes the numbers of mutations per sample shown with corresponding age and gender. Different colors refer to mutational types and ages at low panel.

The burden of coding mutations across the 31 OTS varied from 7 to 194 mutations per tumor (median=36, **Supplementary Table S2**). Mutational signatures analysis identified prevalent signature 5 (cosine similarity = 0.75). Signature 5 was known to be associated with age-related mutational process (28). Out of 31 OTS samples, 27 samples (27/31, 87.1%) harbored at least one genomic alterations at exome scale, listed in the mutational landscape (**Figure 1**). *TP53* mutations were seen in patients aged 10 years and over, while *MYC* mutations occurred in patients aged ranged from 10 to 30 years, and *CDK6* mutations in patients aged less than 20 years. All somatic coding alterations were summarized in **Supplementary Table S2**. We used four algorithms to define SMGs and considered as positive SMGs with at least two methods detected (**Supplementary Table S3**). TP53, RB1, and FOXO3 were identified to SMGs (**Figure 1**).

**Figure 2:**
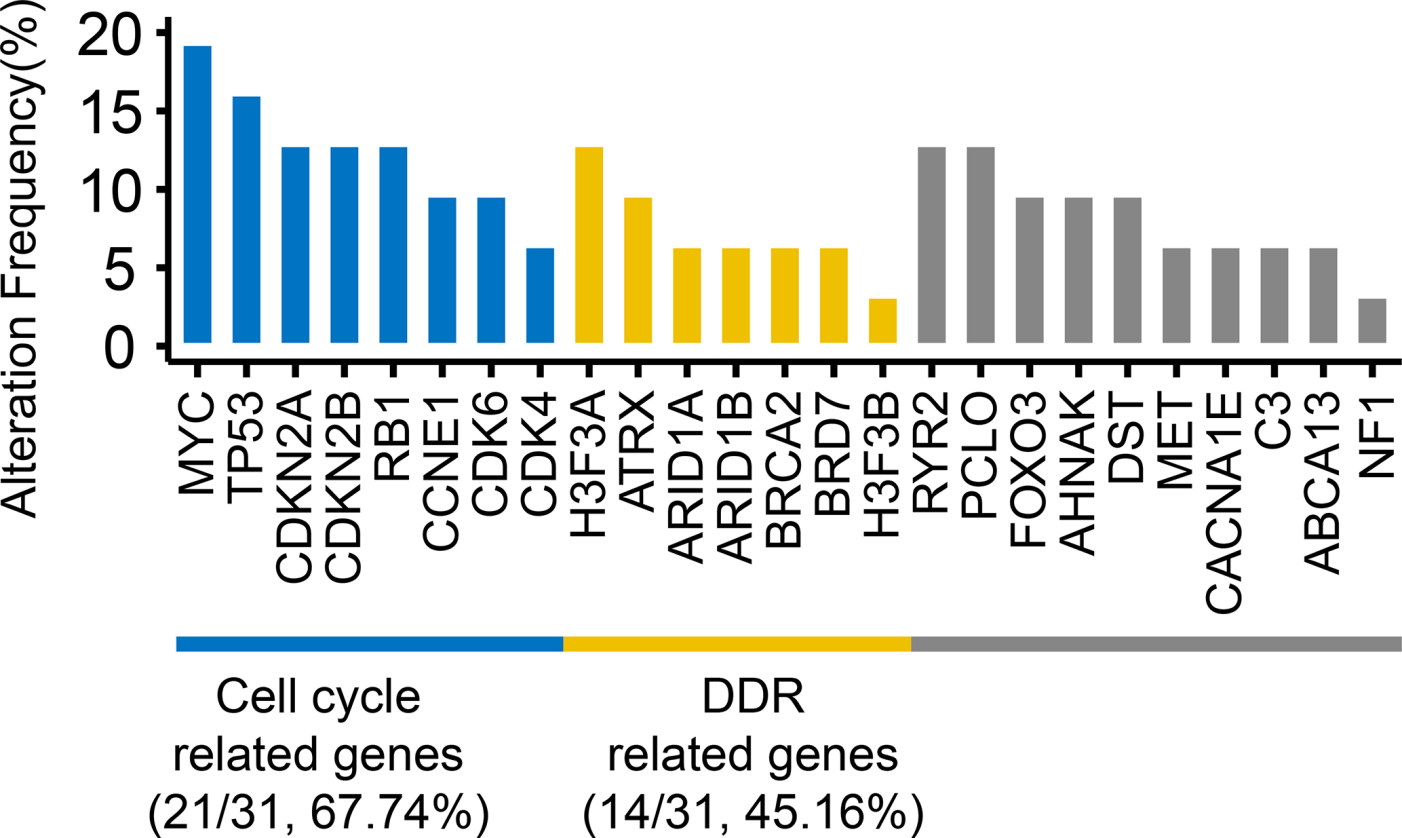
The frequencies of top mutated genes. The genes with the mutations occurring ≥4 samples and reported driver genes were included. Numbers in parentheses represented the number and frequency of mutated samples.

The most frequent genetic alterations were shown in **Figure 2**. Among these mutated genes, 83.87% (26/31 samples) of the mutated genes were mainly divided into two groups (**Figure 2**): cell cycle (21/31 samples, 67.74%) and DDR-related genes (14/31 samples, 45.16%). Cell cycle-related genes included *TP53* (16.13%), *MYC* (19.35%), *CDKN2A* (12.9%), *CDKN2B* (12.9%), *CDK6* (9.68%), *CDK4* (6.45%), *RB1* (12.9%), and *CCNE1* (9.68%). DDR-related genes included *ATRX* (9.68%), *H3F3A* (12.9%), *H3F3B* (3.23%), *ARID1A* (6.45%), *ARID1B* (6.45%), *BRCA2* (6.45%), and *BRD7* (6.45%). Besides, *RYR2* (12.9%) and *CACNA1E* (6.45%) are Ca^2+^ channel-related genes. Piccolo presynaptic cytomatrix protein (*PCLO*, 12.9%) is involved in establishing active synaptic zones and in synaptic vesicle trafficking.

Significant fraction of mutated genes in our cohort belongs to DDR and cell cycle related pathways, consistent with published literature (28). We wonder whether any of mutated gene or group of samples with deficiency in specific pathway correlates with survival of OTS patients. Univariate survival analysis showed that neither of these factors in our cohort was associated with PFS or OS (**Supplementary Table S4**).

We wonder whether any of recurrent copy number amplification or deletion event correlates with survival of OTS patients. Recurrent CNV events were selected in 5 samples at least. Univariate survival analysis showed that amplification region 17p12, 21q22.11, deletion region 8q24.3, 20q13.33 was associated with PFS, deletion region 7p22.3 was associated with OS (**Table 2**, **Supplementary Table S5**).

**Table 2:** Association between recurrent CNV events and patient’s survival.

Genome region	Genomic boundary	PFS			OS		
		HR	95% CI	P-value	HR	95% CI	P-value
17p12 (Amp)	chr17:14248838-15343688	0.60	0.38-0.95	0.02	0.82	0.40-1.65	0.57
21q22.11 (Amp)	chr21:31066214-31971058	0.56	0.36-0.87	0.01	0.75	0.39-1.44	0.38
8q24.3 (Del)	chr8:145831032-145998709	0.28	0.11-0.74	0.01	0.46	0.11-1.92	0.27
20q13.33 (Del)	chr20:59053078-63025520	0.43	0.19-1.00	0.04	0.33	0.08-1.29	0.09
7p22.3 (Del)	chr7:1-330250	0.65	0.27-1.53	0.32	0.27	0.01-1.02	0.04

P-values were calculated by log-rank test.

### TMB-High Is Associated With Improved Progression-Free Survival

**Figure 3:**
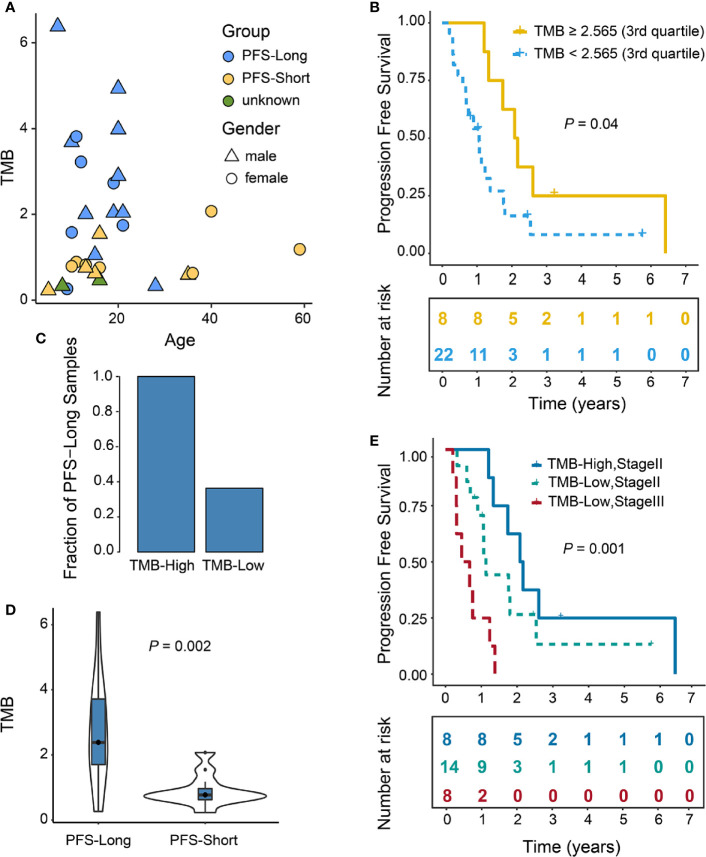
High TMB predicted longer PFS than low TMB. Among 31 patients, 30 patients were included since the clinical information of 1 patient was incomplete. **(A)** The association of TMB with gender and PFS. Different colors represent relative PFS values. The triangle represents males, and the circle represents females. Patients with PFS>400 days were selected in PFS-Long group (n=16), PFS<400 days selected in PFS-Short group (n=12), two patients with incomplete PFS information selected in unknown group (n=2). **(B)** Improved PFS in patients with high TMB. TMB was stratified with 3^rd^ quartile as cutoff=2.565. *P* value was calculated using log-rank test (*P* = 0.04). **(C)** The percentage of PFS-Long in TMB-High (n=8) and TMB-Low (n=22) groups (*P* = 0.003, Fisher’s exact test). **(D)** Violin plot comparing the difference in TMB between PFS-Long (n=16) and PFS-Short (n=12) groups. *P* value was calculated in two tailed Mann Whitney U test (*P* = 0.002). **(E)** Improved PFS in patients with high TMB and Stage II. Patients were stratified by the combination of Stage and TMB. TMB-High-Stage II (n=8), TMB-High-Stage III (n=0), TMB-Low Stage II (n=14), TMB-Low Stage III (n=8). P-value was calculated using log-rank test (*P* =0.001). The numbers in parentheses represent the number of patients in each group.

To characterize TMB in OTS patients, we first analyzed the distribution pattern of TMB (**Figure 3A**), correlation analysis indicated that TMB was not associated with age (*ρ*= 0.10, *P*=0.59). Secondly, all patients with PFS-Short (n=12, yellow) had low TMB. Since high TMB is associated with PFS in several cancer types [4~10], we investigated whether high TMB was associated with PFS of patients with OTS. We defined TMB ≥2.565 (3^rd^ quartile) as TMB-High and <2.565 as TMB-Low (9). The mean PFS of TMB-High versus with TMB-Low was 947.75 (435.54–1459.96, 95% CI) versus 450.73 (256.97–644.49, 95% CI) days, and the median was 775.5 (598-1007.25, 1^st^–3^rd^ quartile) versus 351 (223.5-489.5, 1^st^–3^rd^ quartile) days. TMB-High was positively associated with PFS (*P* = 0.04) (**Figure 3B**). Moreover, the fraction of PFS-Long (defined as the survival length >400 days) was higher in TMB-High group than that in TMB-Low group (100% versus 36.4%) (*P*=0.003) (**Figure 3C**). To investigate the possibility of the patients in PFS-Short group had high TMB values, we compared their TMB values in PFS-Long and -Short groups. As illustrated in **Figure 3D**, no TMB values in PFS-Short group were greater than 2.565, indicating that patients in PFS-Short group had low TMB values.

When evaluating the potential of candidate biomarker on predicting patients’ survival, confounders may be involved and lead to biased conclusion. We therefore tested each potential cofounder including tumor stage, gender, age and surgical margins wide resection (SMWR) in univariate analysis followed by a multivariate Cox model (**Table 3**). We found that the stage II is the only variate associated with prolonged PFS, and all patients with TMB-High are stage II, which could explain that TMB is not significantly associated with PFS when taking stage as a covariate (**Table 3**). To further dissect the effect of TMB and stage on PFS, we divided patients into different groups based on the combination of TMB and stage (**Figure 3E**). Significant difference on PFS was found among these groups (P =0.001).

**Table 3:** Analysis of factors associated with PFS.

Variable	Univariate analysis			Multivariate analysis		
	HR	95% CI	P-value	HR	95% CI	P-value
TMB ≥ 2.565	0.38	0.15–0.99	0.04	0.53	0.19–1.49	0.23
Age ≥ 18	0.78	0.34–1.77	0.55			
Male	0.65	0.29–1.47	0.30			
Stage diagnosis II	0.19	0.07–0.51	0.0002	0.24	0.09–0.68	0.01
Surgical margins wide resection (SMWR)	0.57	0.25–1.3	0.17			

P-value was calculated using log-rank test.

### TMB-High Independently Predicts Improved Overall Survival

**Figure 4:**
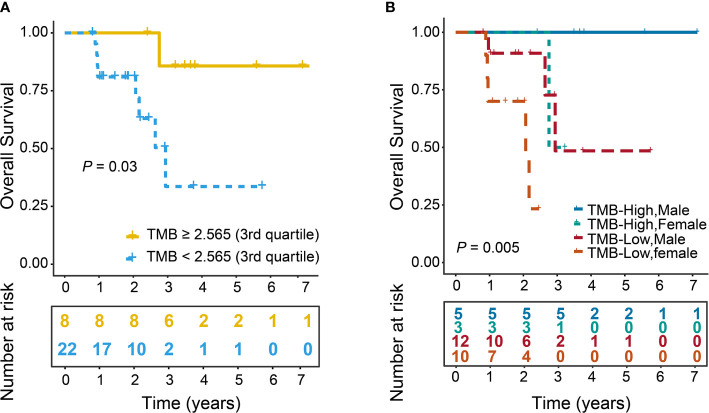
Improved OS in patients with high TMB. TMB was stratified with a binary cutoff of 2.565 (3rd quartile). Log-rank test was used to calculate *P* value. **(A)** OS curves of TMB-High and TMB-Low groups (*P* = 0.03). **(B)** OS curves of patients stratified by the combination of Gender and TMB. TMB-High Males (n=5), TMB-High Females (n=3), TMB-Low Males (n=12), TMB-Low Females (n=10) (*P*=0.005). The numbers in parentheses represented the number patients in each group.

We further investigated the correlation of TMB with OS. The mean OS of TMB-High versus TMB-Low was 1462.75 (981.28–1944.22, 95% CI) versus 723.77 days (535.42–912.13, 95% CI), and the median was 1307 (1133.5–1548, 1^st^–3^rd^ quartile) versus 672.5 days (379.5–871.25, 1^st^–3^rd^ quartile). In addition, we found that TMB-High was significantly associated with improved OS (*P*=0.03, **Figure 4A**). Furthermore, we further tested each potential clinical cofounder including tumor stage, gender, age and SMWR in univariate analysis followed by a multivariate Cox model. We found that TMB-High and male were shown to be able to independently predict improved OS (*P*=0.03, *P*=0.02, **Table 4**, **Supplementary Figure 1**). Moreover, we divided patients into different groups based on the combination of TMB and Gender. Interestingly, survival analysis showed that TMB-High male patients had the best OS, and TMB-Low male patients had better OS than TMB-Low female patients (*P*=0.005, **Figure 4B**). Collectively, our results indicate that both TMB-high and male were associated with prolonged OS.

**Table 4:** Analysis of factors associated with OS.

Variable	Univariate analysis			Multivariate analysis		
	HR	95% CI	P-value	HR	95% CI	P-value
TMB ≥ 2.565	0.14	0.02–1.12	0.03	0.05	0.004–0.73	0.03
Age ≥ 18	0.79	0.21–3.02	0.73			
Male	0.20	0.05–0.84	0.02	0.07	0.008–0.67	0.02
Stage diagnosis II	0.35	0.08–1.56	0.15			
Surgical margins wide resection (SMWR)	1.10	0.29–4.10	0.89			

P-value was calculated using log-rank test.

In addition, SMWR had better local recurrence free survival than positive margin resection in OTS patients (29), therefore we analyzed that whether SMWR was associated with PFS and OS. Our results showed that SMWR was not associated with PFS and OS (**Table 3**, **Table 4**), but slightly significantly associated with long PFS (*P*=0.05) only in TMB-Low group (**Supplementary Table S6**), suggesting that SMWR might be a good therapy strategy for TMB-Low patients with OTS.

Lastly, we also tested the association between driver mutations and TMB. However, our statistical analysis did not find positive correlation (**Supplementary Table S7**).

## Discussion

Predicting clinical outcomes of patients with OTS initially receiving chemotherapy is challenging. This is due to complex factors, such as rarity, tumor heterogeneity, lack of actionable driver events, poorly understood pathophysiology. Increasing studies show that High-Throughput Sequencing (HTS) technology enables comprehensive genomic profiling and better understanding of molecular basis of oncology. In this study, we depicted the genomic profile of patients with initially treated OTS. Gene alterations of these patients were mainly enriched in cell cycle-related genes (*TP53*, *MYC*, *CDKN2A*, *CDKN2B*, *CDK6*, *CDK4*, *RB1*, and *CCNE1*) and DDR-related genes (*ATRX*, *H3F3A*, *H3F3B*, *ARID1A*, *ARID1B*, *BRCA2*, and *BRD7*) as well as Ca^2+^-related genes (*RYR2*, *CACNA1E* and *PCLO*). Moreover, we demonstrated that high TMB (defined as ≥2.565) can be used as an independent prognostic marker in OTS patients.

Most of mutated genes in this study were reported in previous studies. MDM2 was not altered in our cohort, similar to the TP53, CDKN1A, or CDK4 whose alterations were missing in some OTS patients (30–32), indicating that the molecular alterations of OTS is diverse. Besides, we first reported that PCLO mutations were occurred in 12.9% of patients. PCLO is associated with active synaptic zones and synaptic vesicle trafficking (33, 34). Recent studies revealed that PCLO regulates protein ubiquitination and proteasome-mediated proteolysis (35). Although the role of PCLO in cancer is unclear, it is the fifth most frequently mutated gene in esophageal squamous cell carcinoma (ESCC) (36), which is consistent with our study. PCLO overexpression is associated with lymph node metastasis, poor OS and poor disease free survival (DFS) in ESCC (37). PLCO expression promotes the development of ESCC *via* upregulating EGFR signaling (37). EGFR signaling contributes to chemotherapy resistance in OTS (38). Therefore, it is plausible that PCLO mutations might enhance chemotherapy response of patients with OTS.

TMB has been shown to increase with patients’ age from 10 to 90 years old (39). In contrast, our result showed that TMB was independent of age. Compared with most of cancer types, TMB in OTS is relatively low. Therefore, it is not surprising that the correlation between TMB and age was not found.

Our study showed that all patients with TMB-High (n=8) were PFS-Long (n=8), while 36.4% of patients with TMB-Low (n=22) were PFS-Long (n=8). In addition, TMB is significantly greater in PFS-Long than those in PFS-Short. In line with our findings that TMB-High was significantly associated with improved PFS, high TMB predicted prolonged PFS in breast cancer (4) and some cancer types (5–8, 40) Moreover, we found that TMB-High was independently associated with OS, consistently with findings in previous studies conducted in breast cancer, lung cancer and melanoma (4–7, 40). In addition, our results also showed that male was associated with improved OS. Besides, we found that SMWR was correlated with long PFS only in patients with TMB-Low, consistent with the study that SMWR was associated with long PFS in OTS patients (29).

High TMB has also been shown to predict worse PFS and OS in RMS (8), Ewing sarcoma (9), neuroblastoma (41) and resectable pancreatic cancer (42). TMB has a controversial association with PFS and OS across different cancer types due to several possible reasons. First, cancer is highly heterogeneous. The molecular characteristics at genomic, transcriptomic as well as epigenetic level varies significantly across different cancer types. Somatic mutation burden as only one oncogenic signature poses limited influence on tumor progression. Second, different studies apply their own quantitative criteria when stratifying patients based on TMB. The lack of TMB quantification standard may lead to biased analytical results (8–11, 40–42). Lastly, technical variation such as sampling bias during sample extraction and downstream experimental procedure may lead to biased TMB quantification as well.

The relatively low TMB, the small sample size and all samples from one single institution in our study restrain the power to evaluate the impact of the gene alterations in prognosis. In the future, we aim to expand our cohort by recruiting new patients with OTS and leveraging publicly available datasets. Given that PCLO in regulating EGFR signaling in ESCC and high frequency of its mutations in OTS, we will investigate whether PCLO plays a role in the development of OTS and chemotherapy response.

In conclusion, TMB can be used to predict the prognosis for OTS. It might be utilized to predict the clinical outcomes at diagnosis with other risk factors and guide treatment decisions for patients with OTS.

## Data Availability Statement

The raw data has been uploaded onto National Genomics Data Center of China. The accession code is CNP0001734.

## Ethics Statement

The studies involving human participants were reviewed and approved by Medical Ethics Committee of Peking University People’s Hospital. Written informed consent to participate in this study was provided by the participants’ legal guardian/next of kin. Written informed consent was obtained from the individual(s), and minor(s)’ legal guardian/next of kin, for the publication of any potentially identifiable images or data included in this article.

## Author Contributions

Conception and design: LX, YFY, and WG. Provision of study materials or patients: WG, XT, YY, and TJ. Collection and assembly of data: LX, JX, and XS. Laboratorial work and molecular biological analysis of this study: KL and TR. Data analysis and interpretation: LX, DC, and YFY. Manuscript writing: LX. Final approval of manuscript: LX, YFY, WG, DC, JX, XS, KL, TR, XL, YY, TJ, and XT. Accountable for all aspects of the work: LX, YFY, WG, DC, JX, XS, KL, TR, XL, YY, TJ, and XT. All authors contributed to the article and approved the submitted version.

## Funding

This study was supported by Chinese National Natural Science Foundation (No. 81572633 and 81972509) and the Research and Development Fund of Peking University People’s Hospital (Clinical Medicine +X Cultivation Project, No. RDX2019-08).

## Conflict of Interest

YFY and DC are the employees of Genetron Health (Beijing) Co. Ltd.

The remaining authors declare that the research was conducted in the absence of any commercial or financial relationships that could be construed as a potential conflict of interest.

## Supplementary Material

Supplementary Figure 1 OS curves of Male and Female groups. P-value was calculated using log-rank test (*P* = 0.02).

## Abbreviations

OTS, osteosarcoma; PCLO, Piccolo presynaptic cytomatrix protein; TMB, tumor mutational burden; WES, whole exosome sequencing; PFS, progression-free survival; DFS, disease-free survival; OS, overall survival; NGS, Next generation sequencing.
