# Supplementary figures and images for: The Clinical Implications of Tumor Mutational Burden in Osteosarcoma
Source: Front Oncol. 2021 Apr 7;10:595527. doi: 10.3389/fonc.2020.595527 (PMC8059407; doi:10.3389/fonc.2020.595527)

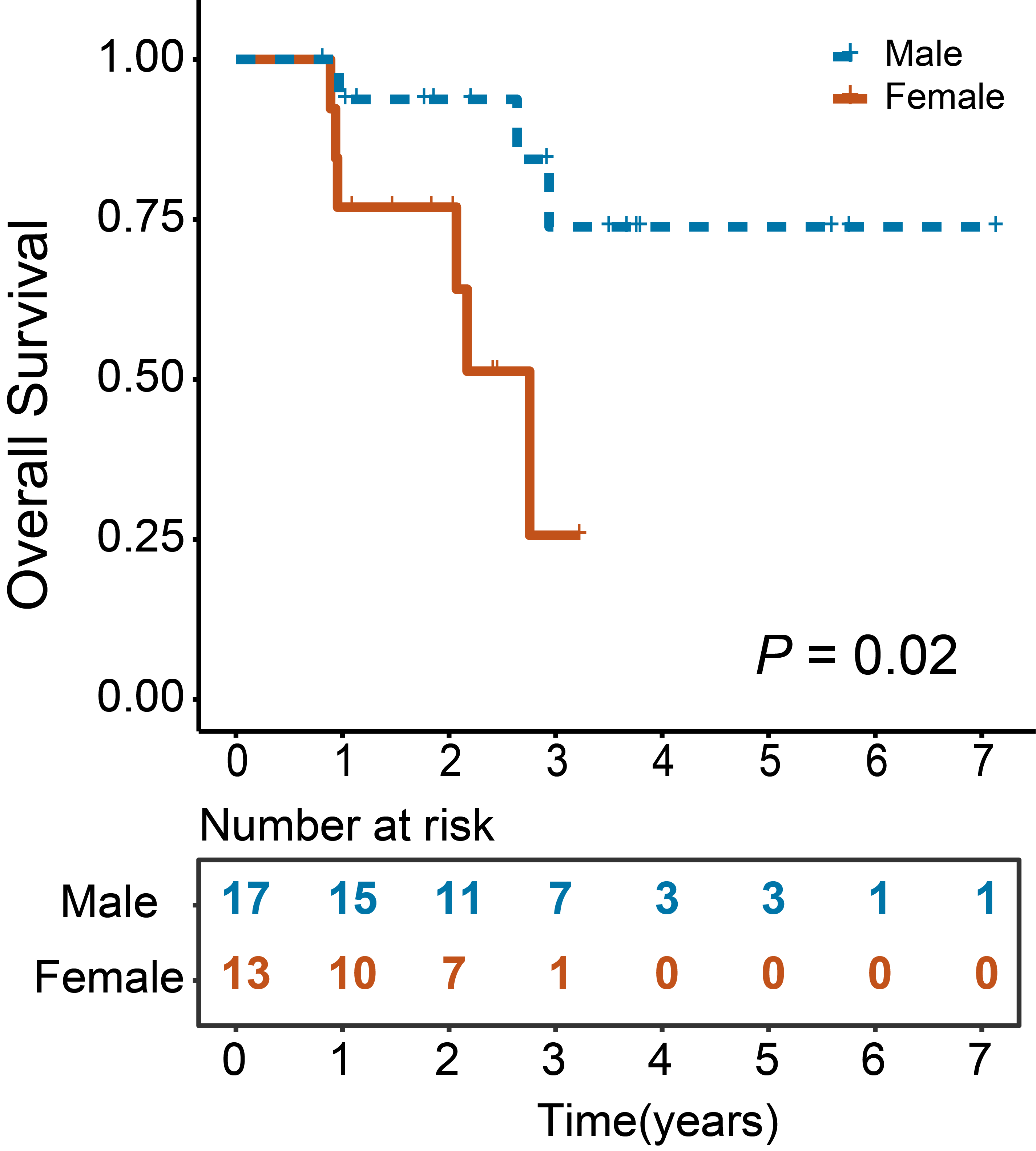

Supplement: Supplementary Figure 1 — OS curves of Male and Female groups. P-value was calculated using log-rank test (P = 0.02). [file Image_1.tif]
