# Supplementary material for: The Clinical Implications of Tumor Mutational Burden in Osteosarcoma
Source: Front Oncol. 2021 Apr 7;10:595527. doi: 10.3389/fonc.2020.595527 (PMC8059407; doi:10.3389/fonc.2020.595527)
Supplement: Supplementary file 5 [file Table_4.docx]

**Supplementary Table S4 Univariate analysis of mutated genes associated with PFS and OS**

|  |  | PFS |  |  | OS |  |
| --- | --- | --- | --- | --- | --- | --- |
| Genomics variable | HR | 95% CI | P-value | HR | 95% CI | P-value |
| *TP53* | 1.45 | 0.49-4.3 | 0.5024 | 2.079 | 0.4-11 | 0.3747 |
| *MYC* | 0.6297 | 0.21-1.9 | 0.4056 | 1.117 | 0.23-5.5 | 0.8917 |
| *PCLO* | 0.3393 | 0.079-1.5 | 0.1276 | 3.727e-09 | 0-Inf | 0.1453 |
| *CDKN2B* | 0.3758 | 0.086-1.6 | 0.1776 | 4.308e-09 | 0-Inf | 0.2252 |
| *CDKN2A* | 0.3758 | 0.086-1.6 | 0.1776 | 4.308e-09 | 0-Inf | 0.2252 |
| *H3F3A* | 1.945 | 0.55-6.9 | 0.2926 | 1.267e-08 | 0-Inf | 0.2981 |
| *DST* | 0.3758 | 0.086-1.6 | 0.1776 | 4.308e-09 | 0-Inf | 0.2252 |
| *CDK6* | 1.745 | 0.39-7.8 | 0.4588 | 1.266e-08 | 0-Inf | 0.4836 |
| *RB1* | 1.633 | 0.55-4.8 | 0.3721 | 4.348e-09 | 0-Inf | 0.2199 |
| *F8* | 3.095 | 0.39-25 | 0.2618 | 4.333 | 0.5-37 | 0.1461 |
| *DNAH11* | 0.5079 | 0.12-2.2 | 0.3535 | 0.9096 | 0.11-7.4 | 0.9292 |
| *RYR2* | 1.072 | 0.36-3.2 | 0.9005 | 4.222e-09 | 0-Inf | 0.1971 |

The types of mutations were SNV/InDel/CNV. Genes with mutations ≥4 and reported driver genes were selected in Table 2. *P* values were calculated by log-rank test.
