# Supplementary material for: The Clinical Implications of Tumor Mutational Burden in Osteosarcoma
Source: Front Oncol. 2021 Apr 7;10:595527. doi: 10.3389/fonc.2020.595527 (PMC8059407; doi:10.3389/fonc.2020.595527)
Supplement: Supplementary file 7 [file Table_6.docx]

**Supplementary Table S6. Analysis of Surgical margins factors associated with PFS and OS in TMB-Low group**

|  | PFS | | | OS | | |
| --- | --- | --- | --- | --- | --- | --- |
| Variable | HR | 95% CI | P-value | HR | 95% CI | P-value |
| Surgical margins wide resection (SMWR) | 0.36 | 0.13-1.04 | 0.05 | 0.43 | 0.09-1.98 | 0.27 |

P-values were calculated by log-rank test
